# Heterogeneity of clinical features and corresponding antibodies in seven patients with anti-NMDA receptor encephalitis

**DOI:** 10.3892/etm.2015.2689

**Published:** 2015-08-19

**Authors:** KURT-WOLFRAM SÜHS, FLORIAN WEGNER, THOMAS SKRIPULETZ, CORINNA TREBST, SAID BEN TAYEB, PETER RAAB, MARTIN STANGEL

**Affiliations:** 1 Department of Neurology, Hannover Medical School, Hannover D-30625, Germany; 2 Department of Neuroradiology, Hannover Medical School, Hannover D-30625, Germany

**Keywords:** anti-N-methyl-D-aspartate receptor antibodies, cerebrospinal fluid, encephalitis

## Abstract

Anti-N-methyl D-aspartate (NMDA) receptor encephalitis is the most common type of encephalitis in the spectrum of autoimmune encephalitis defined by antibodies targeting neuronal surface antigens. In the present study, the clinical spectrum of this disease is presented using instructive cases in correlation with the anti-NMDA receptor antibody titers in the cerebrospinal fluid (CSF) and serum. A total of 7 female patients admitted to the hospital of Hannover Medical School (Hannover, Germany) between 2008 and 2014 were diagnosed with anti-NMDA receptor encephalitis. Among these patients, 3 cases were selected to illustrate the range of similar and distinct clinical features across the spectrum of the disease and to compare anti-NMDA antibody levels throughout the disease course. All patients received immunosuppressive treatment with methylprednisolone, intravenous immunoglobulin and/or plasmapheresis, followed in the majority of patients by second-line therapy with rituximab and cyclophosphamide. The disease course correlated with NMDA receptor antibody titers, and to a greater extent with the ratio between antibody titer and protein concentration. A favorable clinical outcome with a modified Rankin Scale (mRS) score of ≤1 was achieved in 4 patients, 1 patient had an mRS score of 2 after 3 months of observation only, whereas 2 patients remained severely impaired (mRS score 4). Early and aggressive immunosuppressive treatment appears to support a good clinical outcome; however, the clinical signs and symptoms differ distinctively and treatment decisions have to be made on an individual basis.

## Introduction

Encephalitis with prominent neuropsychiatric symptoms and post-mortem evidence of inflammatory lesions was described in the 1960s as limbic encephalitis (LE) (1). Subsequent studies identified an association between LE and antibodies directed against tumor and brain tissue, establishing LE as a paraneoplastic disease. Typical clinical features include disturbance of consciousness, short-term memory, psychosis and seizures. The antibodies are directed against intracellular antigens and many of these onconeuronal antibodies are associated with certain malignancies, such as small cell lung cancer. Cancer is typically diagnosed in ≥95% of patients with these antibodies (2) (Table I). However, it is unlikely that these antibodies are directly pathogenic due to their intracellular targets. Antibody transfer (anti-Yo) failed to provoke respective histopathological or typical clinical features (3), and neuronal loss appears to be T-cell driven (4). These antibodies can be divided into three subgroups: Ia, containing the classical onconeuronal antibodies, such as anti-Hu, anti-Yo and anti-Ri; Ib, cancer-associated antibodies (SOX and ZIC) lacking an association with an immune response causing a paraneoplastic syndrome (PNS); and Ic, non-PNS antibodies, including glutamate decarboxylase, associated with cerebellar ataxia. Antibodies in the Ia group are attributed to the majority of paraneoplastic syndromes (PNS) with anti-Hu and anti-Yo as the most common, accounting for up to ~50% of all PNS antibodies (5).

After the year 2000, a second set of autoantibodies was described (6–9), which are directed against surface antigen epitopes, primarily ion channels. In addition to possessing different target antigen locations, these antibodies exhibit a lower coincidence with malignancies, varying from 3% (glycine AB) up to 70% (α-amino-3-hydroxy-5-methyl-4-isoxazolepropionic acid antibodies (AMPA) (2). In anti-NMDA receptor encephalitis, younger patients are at a reduced risk of presenting with a tumor (10). Surface target structures are associated with the voltage-gated potassium channel (VGKC; antibodies LGI1 and Caspr2), ligand dependent ion channels, such as ionotropic glutamate receptors antibodies (NMDA, AMPA), GABA_A_, GABA_B_, and glycine receptor antibodies. Present in ~4% of all autoimmune-mediated encephalitis, the anti-NMDA receptor is the most common. Large case series studies involving >200 patients have been published on the disease course, therapeutic intervention and role of the NMDA receptor antibody (11–13). A direct pathogenic role of these antibodies can be assumed, as immunotherapy mitigates the clinical symptoms and improves neurological outcome (11,14–16). Furthermore, cell-culture experiments have shown a reversible downregulation of NMDA receptors on antibody-exposed cells, mediated by titer-dependent capping and internalization. Thereby the surface expression of the NMDA receptor is diminished prominently, affecting the temporal lobes and hippocampus (12) and disturbing cell communication (for example, GABAergic and dopamine) pathways. Neuropsychiatric symptoms are the common clinical feature of anti-NMDA receptor encephalitis. The distinct involvement of the peripheral nervous system may be explained by the varying expression patterns of the surface antigen (17); for example, neuromyotonia is more common in Caspr2- compared with LGI1-mediated disease. Anti-NMDA receptor antibodies are directed against an extracellular epitope of the Glu-N1 unit of the NMDA receptor. To date, 7 different subunits in 3 subfamilies (Glu-N1, Glu-N2a-d and Glu-N3a+b) have been identified. The NMDA receptor is a heterotetrameric assembly of an NR1 subunit, usually in combination with modulatory NR2 and/or NR3 units. The NR2 subunit composition primarily defines the gating and functional differences of the receptor (18,19). Its composition varies between cell type (neuron, oligodendrocyte, astrocyte) and site of expression (20–23).

## Materials and methods

### 

#### Patients

The present study included 7 patients with anti-NMDA receptor encephalitis admitted to the hospital of Hannover Medical School (Hannover, Germany) between 2008 and 2014. This study was approved by the local Ethics Committee of Hannover Medical School and patients or their carers provided written informed consent. The details of patient 1 have been previously reported in part (24) and more recently in association with brain metabolic changes in autoimmune encephalitis (25).

#### Testing and diagnosis

Routine blood tests included blood count and measurement of serum electrolytes, liver enzymes, creatine kinase (CK), glucose, partial thromboplastin time (PTT) and international normalized ratio (INR). Cerebrospinal fluid (CSF) was available for extraction in 6 patients, and subjected to a variety of analyses, including cell count, glucose, lactate, total protein, albumin, IgG, IgA, IgM, oligoclonal bands, and microbiological and virological analysis. NMDA receptor IgG antibodies were detected by immobilizing human embryonic kidney cells (HEK 293) transfected with the NR1 subunit of the NMDA receptor on BIOCHIPS (Euroimmun AG, Lübeck, Germany), and incubating them with patient sera and/or cerebrospinal fluid. Primary dilutions were 1:10 in serum and 1:1 in CSF. Quantification was obtained via the specific fluorescence intensities using an indirect immunofluorescence test, and expressed as end-point titers. Patients were diagnosed with anti-NMDA receptor encephalitis when presenting typical clinical features in combination with CSF and/or serum NMDA receptor IgG antibodies. Magnetic resonance imaging (MRI) fields, including contrast application, were obtained using a 1.5 or 3.0-Tesla MRI instrument. Clinical outcome was rated using the modified Rankin scale (mRS).

## Results

### 

#### Patient admission

A total of 7 female patients admitted to the hospital between 2008 and 2014 were diagnosed with anti-NMDA receptor encephalitis. On admission, patients were aged between 23 and 57 years old. Hospital admission was via the accident and emergency department in 4/7 cases, due to the subacute onset of neuropsychiatric symptoms, including personality changes.

#### Case repor

##### Case 1 (patient 2)

A 34-year-old woman with no pre-existing illness was admitted to the hospital with the subacute onset of personality change, anxiety and psychotic-hallucinatory perception. The results of cranial MRI and thoracic/abdominal computed tomography (CT) examination appeared normal. In the electroencephalography (EEG), a continuous slow activity with an intermittent right temporal focus was observed. Within 2 days after admission, the patient developed orofacial dyskinesia, and severe focal and generalized seizures, which were only interrupted using high doses of phenobarbital. Eventually, the patient developed central hypoventilation with respiratory insufficiency and required ventilation (mRS 5). The results of a fludeoxyglucose positron emission tomography (FDG-PET) examination were unremarkable regarding tumor screening; however, an abdominal ultrasound revealed a suspicious lesion in the right ovary. In response to this observation, an ovariectomy was conducted 9 days after patient admission, which revealed a teratoma. CSF analysis revealed oligoclonal bands without pleocytosis, and anti-NMDA receptor antibodies were detectable in the CSF (1:100) and serum (1:800). In the intensive care unit (ICU), 2 weeks after admission, therapy was initiated with immunoabsorption (6 applications) followed by methylprednisolone (1 g/day for 5 days). However, the patient developed a severe autonomic dysfunction, with cardiac arrhythmia, blood pressure dysregulation, disturbed thermoregulation and hypersalivation. EEG and particularly the cardiorespiratory situation worsened to a critical condition within the next month. An additional left ovariectomy was performed, revealing no teratoma, and a second cycle of immunoabsorption (6 applications) followed by 4 cycles of cyclophosphamide (600 mg/m^2^) was administered at monthly intervals. Towards the fourth cycle of treatment, the patient started to improve steadily. The immunosuppressive therapy was de-escalated to immunoglobulins (0.4 g/kg/day for 5 days). Repetitive analysis of CSF and serum NMDA receptor antibodies showed decreasing antibody titers (Table II). Thoracic and abdominal CT was repeated after 6 months, in addition to MRI of the pelvis. After 7 months in the ICU, the patient was discharged for rehabilitation. At this time point the patient was oriented only to herself but had lost orientation in time and space, and showed fluctuating vigilance and cooperation. The patient was followed up for 3 years and her clinical state gradually improved. On the final visit the patient was fully orientated, cooperative and able to look after herself (mRS 1).

###### Case 2 (patient 5)

A 44-year-old woman was referred to the hospital for confirmation of a diagnosis of anti-NMDA receptor encephalitis. Three months previously, the patient had initially been admitted to a different hospital with a right temporal anopsy, preceded by a mild generalized headache and mood changes with latent aggressive behavior. Alpha activity with signs of varying vigilance were described in the EEG. Within a week, the patient developed aphasia with semantic paraphrasia, severe apraxia, anxiety, hallucinations and reduced orientation to person, time, place and situation. The results of a cranial MRI examination were unremarkable. CSF analysis revealed 156 cells/µl, positive oligoclonal bands, a positive Epstein-Barr virus polymerase chain reaction (PCR) analysis and elevated 14-3-3 protein concentrations. Infectious meningoencephalitis was suspected and antiviral and antibiotic therapy was administered. After 1 week, the patient began to develop orofacial dyskinesia. Final results from CSF revealed NMDA receptor IgG antibodies in the CSF and serum (Table II). The patient was administered haloperidol (2×2.5 mg) and prednisolone (100 mg, orally), but subsequently developed a neuroleptic malignant syndrome. The haloperidol was immediately discontinued and the rigor symptoms eased; however, the patient developed a respiratory insufficiency and required ventilation. The patient's clinical condition worsened and after 2 weeks she rapidly developed a trismus-like spasm of the jaw, fracturing several maxillary teeth, and complex focal seizures. The patient was at this point transferred to the hospital at the Hannover Medical School. PET/CT scan results were normal regarding neoplasia; however, due to age and the aggressive clinical symptoms, prophylactic ovariectomy was performed, but revealed no teratoma. The patient was treated with 5 cycles of plasmapheresis, followed by cyclophosphamide (6 cycles of 750 mg/m^2^ in monthly intervals), in combination with rituximab (4 cycles of 375 mg/m^2^ every week for 1 month). The patient was discharged for rehabilitation after 2 months in the hospital. At the time of transfer, the patient exhibited variable vigilance, fluctuating aggression and fecal and urinary incontinence and was fully dependent on assisted care for everyday functions (mRS 4). During the rehabilitative treatment, vigilance and clinical symptoms gradually improved, as the patient relearned everyday skills and her mood stabilized. On the final visit, 21 months after onset, the patient was fully independent and was undergoing work rehabilitation for her job as a translator (mRS 1). As immunosuppressive treatment with cyclophosphamide continued up to 9 months after onset, and an ovariectomy had been performed, no further immunosuppressive treatment was administered, while close clinical monitoring was maintained.

###### Case 3 (patient 6)

A 23 year old woman was admitted via an emergency department due to phasic loss of orientation to time, place and situation, abnormal fearful behavior and stereotypical repetitive movements (mRS 3). Two years previously, the patient had been referred to a psychiatric department with intermittent behavioral disorder. Despite a pleocytosis of 51 cells/µl and oligoclonal bands in the CSF, further encephalitis work-up investigations including cranial MRI and CSF analysis for viral pathogens did not elucidate the cause of the patient's symptoms, which resolved within 6 weeks.

At admission repeated cranial MRI results were normal, as were MRI of the pelvis and PET/CT scans regarding tumor screening. A microinvasive laparoscopy was performed, and ovary biopsy revealed no signs of teratoma. The EEG revealed a slow alpha rhythm with a paroxysmal frontal delta wave focus, suggesting an ictal focus for the stereotype movements, and therefore levetiracetam treatment was initiated (final dose, 2×750 mg). Neuropsychological deficits included memory and executive functions. CSF analysis showed a mild pleocytosis, with 18 cells/µl and oligoclonal bands. NMDA receptor antibodies were tested for only in serum and were found to be positive (1:100). Notably, a serum sample stored from the first episode 2 years previously was analyzed and NMDA receptor antibodies (1:10) were detected. Due to the pleocytosis, acyclovir treatment was administered for 10 days, and after the receipt of the NMDA receptor antibody result 14 days after admission methylprednisolone (1 g/day for 5 days) was administered. The patient's clinical symptoms and EEG results improved rapidly and the patient was discharged after 28 days. However, the patient was readmitted for a planned second course of methylprednisolone (1 g/day for 5 days) 1 month later. At that time, neuropsychological memory deficits remained detectable. A final course of methylprednisolone was administered at 6 months after admission. Towards the final hospital admission, the anti-NMDA receptor antibody concentration decreased and the memory deficits exhibited full remission (mRS 0), enabling the patient to continue an apprenticeship as an industrial management assistant. Due to the mild treatment course, no long term immunosuppressive treatment was initiated.

##### Assessment of CSF

**Figure 1.**
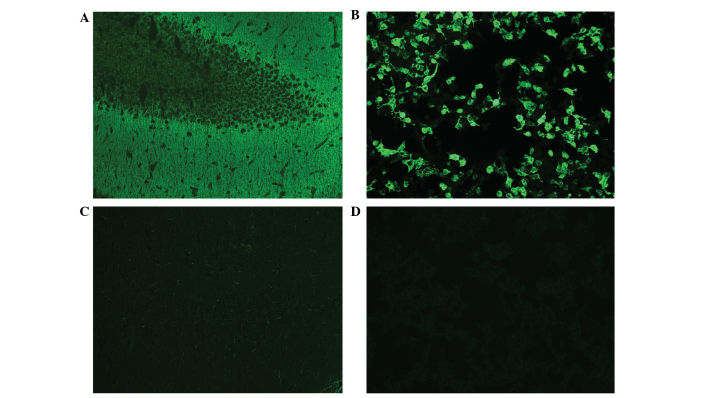
N-methyl D-aspartate (NMDA) receptor immunofluorescence. Patient cerebropsinal fluid (CSF) on a rat hippocampal slide depicting (A) antibody binding on hippocampal neurons and (B) NMDA receptor transfected human embryonic kidney cells incubated with patient CSF, and secondary immunofluorescent antibodies revealing specific antibody binding and (C and D) respective control images.

**Figure 2.**
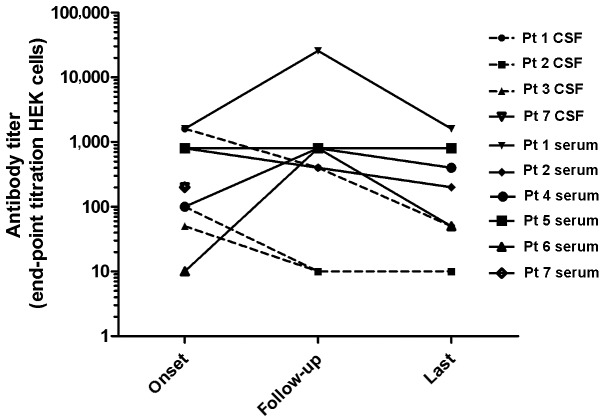
NMDA receptor antibody titers. NMDA receptor antibody end point titration titers in CSF and serum given as specific fluorescence intensities on HEK 293 cells at admission to hospital, follow up (second last) and last examination of antibody titers. Pt, patient.

As a defining condition, anti-NMDA receptor IgG antibodies were found in the CSF and/or serum in all patients. Matching serum/CSF samples could be analyzed in 6 patients. Serum and CSF were screened for NMDA receptor antibodies, followed by end-point titration on transfected HEK 293 cells (Fig. 1). In patient 6, only serum antibody titers were available. In patient 3, NMDA receptor antibodies were only detected in the CSF, which is uncommon but not unusual as serum antibodies are negative in ~15% of patients using detection techniques such as rat brain slice and transfected HEK cells (26). In patients 1 and 2, calculation of the antibody specific index revealed an intrathecal synthesis of antibodies. In patient 4, a matching CSF/serum sample was obtained at the onset of the disease; however, no antibodies were detected in the CSF following repeated analysis. This is a highly unusual observation, as antibodies in the central nervous system (CNS) appear to mediate the clinical symptoms, and patients with anti-NMDA receptor encephalitis without CSF NMDA receptor antibodies have not been described. However, NMDA receptor seropositivity alone has been reported in patients with herpes simplex virus (HSV) encephalitis (13). The highest CSF and serum titers were detected in patient 1, who was one of the two patients that exhibited the least clinical improvement (24,25). Notably, in contrast to the clinical symptoms, the CSF and serum NMDA receptor antibody titer declined between month 10 and 11 during immunosuppressive treatment (Table II). However, simultaneously CSF and serum protein concentrations notably decreased. Therefore, the antibody titer was adjusted by the protein levels to determine the CSF titer/CSF protein and serum titer/serum ratios. As presented in Table II, these ratios increase until the clinical symptoms are alleviated, then decrease according to clinical improvement. In patient 4, the antibody titer in the serum and the ratio serum titer/serum protein increased between admission and the end of follow-up. This patient failed to improve clinically, despite antibody titers in the serum decreasing between months 2 and 7. In patients 2 and 3, the antibody titers and the titer/protein ratio decreased in correlation with clinical improvement. In patients 1, 4, 6 and 7 an increase in antibody titers was detected during the course of the disease (Fig. 2).

##### Additional diagnostic investigations

**Figure 3.**
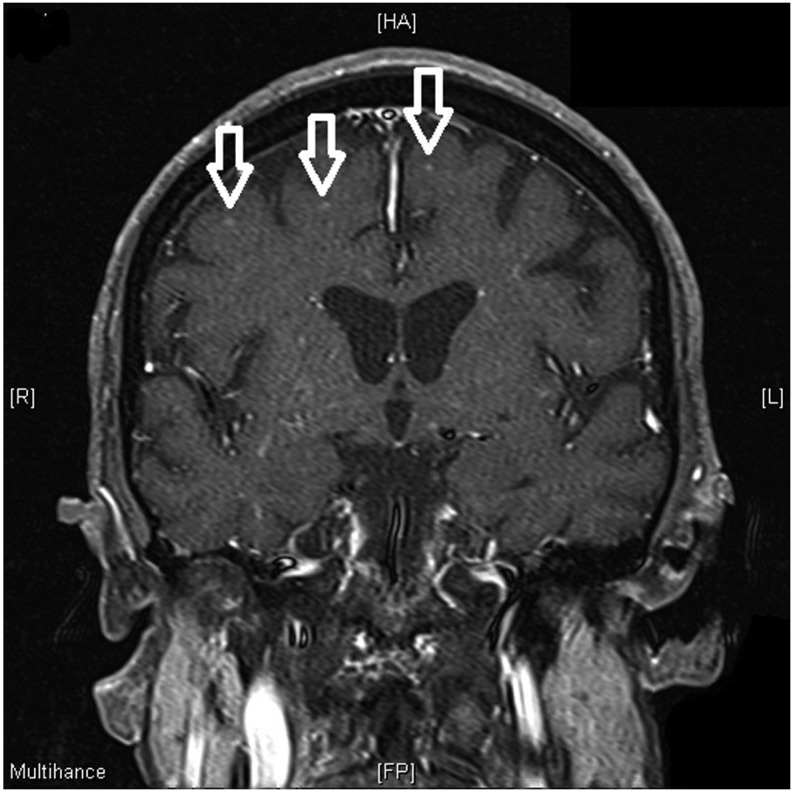
T1-weighted coronal magnetic resonance image of a patient (patient 4) with anti-N-methyl D-aspartate receptor encephalitis and a mRS score of 5 at that time depicting contrast enhancement (white arrows) and subcortical T2-hyper-intensities (T2-weighted image not shown).

MRI scans revealed age-appropriate normal results in 4 patients. In patient 3, a single small unspecific periventricular T2 hyperintense lesion was observed despite severe clinical impairment at that time (mRS 5). In patient 1, hippocampal T2-hyper-intensities and a bilateral diffusion weighted signal elevation were observed. In patient 4 subcortical T2-hyper-intensities with contrast enhancement were noted (Fig. 3). In addition to tumor screening with routine diagnostics, such as chest X-ray and abdominal ultrasound, all patients received FDG-PET. Oophorectomy was performed in patients 1–5. Ovarian teratoma were detected in patients 1 and 2; 90% of tumors associated with this disease are teratomas, with an incidence of 25–56% (11,12,27).

## Discussion

The present case series illustrates the characteristics of the multi-stage clinical presentation and the range of severity of anti-NMDA receptor encephalitis, in context with their anti-NMDA receptor antibodies. The majority of patients were premenopausal women, which is a typical feature of this disease entity; however, anti-NMDA receptor antibodies have been reported in men and women of all ages (28).

In patients with the typical symptoms of sub-acute onset of psychic disturbances, differences in personality, memory loss and seizures, the range of differential diagnoses is limited. Clinical presentation and verification of NMDA receptor antibodies define the diagnosis of anti-NMDA receptor encephalitis. MRI alters only in ~33% of patients, and although EEG and CSF abnormalities are common in 90 and 79% of cases, respectively, the results are unspecific (11). Infectious causes of encephalitis, particularly HSV and less commonly Varicella-Zoster virus or cytomegalovirus, must be excluded via PCR and/or antibody index using CSF examination. Metabolic causes, such as uremic and hepatic encephalopathy, may be assessed by routine laboratory tests. More often, diagnostic uncertainty occurs if symptoms are mild or psychic changes are dominated by negative symptoms, such as depression or avolition, such as during the first episode of patient 6. In young patients, toxic causes due to drug or alcohol abuse with Wernicke's encephalopathy may considered (Table III).

Other autoimmune diseases, such as steroid responsive encephalopathy associated with autoimmune thyroiditis (SREAT, formerly known as Hashimoto encephalopathy) and CNS vasculitis, may be considered as possible differential diagnoses. For example, in patient 4, who presented with subcortical T2-hyperintensities and contrast enhancement in MRI, a vasculitis was suspected and excluded as a possibility by brain biopsy. Considering SREAT, thyroid peroxidase antibodies are identified in 5% of healthy individuals and clinical symptoms respond rapidly to steroid treatment. In older patients the rapid mental deterioration of Creutzfeldt-Jacob disease may resemble the neuropsychiatric symptoms of anti-NMDA receptor encephalitis; however, elevated levels of 14-3-3 protein may be present in the CSF in both diseases.

Commonly in anti-NMDA receptor encephalitis the clinical presentation passes through different stages. During the first stage, the prodromal stage which precedes the next stage by ~2 weeks, 70% of patients suffer from unspecific flu-like symptoms, including fever, headache, nausea and unrest (12,28). In the second phase, neuropsychiatric symptoms are overt and seizures occur. All of the present patients exhibited psychological abnormalities, ranging from unrest and disorientation to fear, affective disturbances and manifested psychosis, hallucinations and loss of short-term memory. Furthermore, all the present patients suffered from generalized or focal seizures. This is in accordance with the prior literature, as psychiatric disturbances are a defining symptom of the disease and the incidence of seizures is 70–80% (12,27). In particular, the symptom combination of neuropsychiatric abnormalities and generalized seizures is distinct from that of other types of autoimmune encephalitis, such as anti-LGI1 encephalitis, in which neuropsychiatric symptoms are less common and mild and predominantly focal seizures occur. Central hypoventilation is another common feature of the early stages of anti-NMDA receptor encephalitis.

The third phase, after 10–20 days (27), is defined by dyskinesias and vegetative dysregulation. Patients 1–3 suffered from autonomic dysfunction with cardiac arrhythmia, blood pressure deregulation, disturbed thermoregulation and hypersalivation.

The initial symptoms of anti-NMDA receptor encephalitis are unspecific flu-like symptoms that usually precede the neuropsychiatric manifestations by 1–2 weeks. This indicates a rapid onset of antibody production, which resembles infectious or acute onset fast-progressing autoimmune diseases such as acute demyelinating encephalomyelitis. Potential associations have been indicated between NMDA receptor antibody production and *Mycoplasma pneumoniae* (29) and HSV, possibly by autoantigen presentation following neuronal cell death (13). A study demonstrated that in a small subgroup of patients, the presence of NMDA receptor antibodies is associated with aquaporin 4 and myelin oligodendrocyte glycoprotein antibodies, which are also observed in patients with neuromyelitis optica or other demyelinating syndromes, suggesting connected immune processes (30). Other than the association with ovarian teratoma, the reason for the predominance of women among patients with anti-NMDA receptor encephalitis remains unclear; however, it is possible that estrogen via stimulated antibody production may be involved (31).

In animal experiments it has been demonstrated that the injection of NMDA receptor antibodies in rats causes corticomotor hyperexcitability (32), which may be the underlying cause of the agitation and seizures observed in the early stage of the disease. Internalization of NMDA surface receptors decreases the neuronal excitability of GABAergic neurons, which highly express NMDA receptors; therefore, it has been speculated that positive symptoms such as dyskinesias are generated by disinhibiting extrapyramidal networks (10,33). It has been hypothesized that the disruption of the blood-brain barrier is a relevant factor for anti-NMDA receptor associated neuropsychiatric diseases (34). Furthermore, it has been suggested that the central hypoventilation observed in patients with anti-NMDA receptor encephalitis may be caused by the disruption of ponto-medullary respiratory reflexes, as occurs in an NMDA receptor blockade (35). Therefore, the time-course of symptoms supports a primarily cortical/hippocampal mechanistic pathway, with secondary subcortical alteration and disturbance of corticostriatal/brainstem pathways. The fact that the NMDA receptor is expressed in hippocampal, cortical and cerebellar neurons, in addition to glial cells such as oligodendrocytes and astrocytes, in varying concentrations and subunit composition may be of further pathophysiological relevance to the clinical presentation and time course of the disease (18,21,23,36,37. The expression varies under different pathophysiological conditions, such as ischemia (38). Memory deficits, as a typical feature of anti-NMDA receptor encephalitis, may be explained by the increased expression of the NMDA receptor in hippocampal neurons (12,39).

It has been demonstrated that the titer of NMDA receptor antibodies correlates with clinical outcome, and that high antibody titers are more common in patients with poor outcome or tumors (12,26). In the present case series, the highest antibody titer was detected in a patient with occult teratoma who failed to be detected by ultrasound, CT, MRI and PET. High antibody titers may be an indicator of an underlying tumor, particularly if there is no decline following immunosuppressive treatment. Despite the correlation between antibody titers, severity of symptoms and the antibody-driven pathogenesis, a number of patients, such as patients 1 and 4 in the patient case series and others in the literature (12,26), failed to improve despite decreasing antibody titers. As shown in the present cases, NMDA receptor antibodies are part of the overall protein concentration measured in the CSF or serum. Reduced antibody titers may therefore correlate with an overall reduction in protein concentration. This is relevant for patients with anti-NMDA receptor encephalitis, since plasma exchange or immunoabsorption in particular, but also ICU treatment and a long-lasting catabolic state significantly decrease the protein concentration in serum and CSF (Table II). This may be avoided by calculating the antibody/protein ratio as opposed to the antibody titers alone. This also elucidates the importance of follow-up CSF analysis as a marker for disease activity and prognosis during the course of this disease and other types of autoimmune and infectious encephalitis (40,41).

The relevance of IgA and IgM anti-NMDA receptor antibodies remains unclear, as they are present in ~9% of healthy individuals. IgG anti-NMDA receptor antibodies are present in ~1% of healthy individuals only (42). Notably, IgA and IgM are more frequently observed in patients with neuropsychiatric disease and a history of blood-brain barrier disruption (43). Furthermore, an association between anti-NMDA receptor IgA and cognitive dysfunction has been detected (44).

In all patients, with the exception of patient 4, immunotherapy was initiated within 14 days after the onset of psychiatric symptoms. The latter patient was referred to the hospital with unknown encephalopathy for >1 year, receiving no immunotherapy during that time. All patients received intravenous methylprednisolone 1 g/day for 5 days. Further primary treatment was 0.4 g/kg intravenous immunoglobulin (IVIG) per day for 5 days and/or plasmapheresis, and the number of repetitions was symptom-dependent. In 4 patients a second-line therapy consisted of 4 cycles rituximab with 375 mg/m^2^ body surface area (BSM) at weekly intervals in combination with 6 cycles cyclophosphamide 750 mg/BSM at monthly intervals. One patient received only first-line therapy (patient 6), the 2 remaining patients received only cyclophosphamide (patient 2) and only azathioprine (patient 4) as second-line therapies. ICU admission was necessary for 4 patients due to central hypoventilation during the course of the disease.

Treatment outcome can be positively influenced by early treatment initiation, and the use of intensified (second-line) treatment if initial therapy fails (observed in 45% of patients) (11). Treatment failure can be assumed if no sustained improvement is observed 4 weeks after first-line therapy. However, all patients were at least moderately disabled during their disease course, 4 patients had a good clinical outcome (mRS ≤1) at last follow-up. Patient 7 improved from mRS 5 to mRS 2 in 3 months of observation time. However, the 2 remaining patients were relevantly disabled (mRs 4). Possible explanations of poorer response to treatment may include deferred diagnosis with delayed treatment initiation in case 4, and delayed diagnosis of teratoma in case 1, as imaging including FDG-PET and explorative laparotomy with ovarian biopsy yielded negative results. However, the late ovariectomy that confirmed teratoma histologically exhibited limited improvement. Controlled trials are lacking. However, the commonly used and recommended treatment regime, as administered to the majority of the present patients, comprises first line methylprednisolone and IVIG and/or plasmapheresis followed by second line rituximab and/or cyclophosphamide, as this treatment protocol reduces clinical symptoms and the levels of anti-NMDA receptor antibodies. Approximately 12% of patients with anti-NMDA receptor encephalitis suffer from relapses; however, immunosuppressive treatment during the first episode and second line immunosuppressive treatment in patients without a tumor is associated with a lower frequency of relapses (11).

In patients with a new subacute onset of neuropsychiatric symptoms, the differential diagnosis of anti-NMDA receptor encephalitis should be considered and CSF analysis may aid the detection of anti-NMDA receptor antibodies. Although multistage clinical presentation is a common feature of the disease, the severity of symptoms is highly variable. Cranial MRI is typically necessary for differential diagnosis; however, in anti-NMDA receptor encephalitis results are unspecific. Depending on the symptoms, other diagnoses may be excluded (Table III). If anti-NMDA receptor encephalitis is confirmed, an intensive search for tumors, particularly teratomas, is required. Immunosuppressive treatment is required immediately, as a good clinical outcome is associated with early therapy for decreasing anti-NMDA receptor antibody concentrations.

## Figures and Tables

**Table I.** Onconeuronal and neuronal surface antigen antibodies. Clinical syndrome, common associated tumors and rate of tumor diagnosis for onconeuronal antibodies in comparison with surface antibodies.

A, Onconeuronal antibodies				

Antibody	Neurological syndromes	Tumors	Probability of cancer (%)
Hu	Encephalomyelitis, cerebellar degeneration, limbic encephalitis, brainstem encephalitis	SCLC	98
CV2	Encephalomyelitis, chorea, cerebellar degeneration, limbic encephalitis	SCLC	96
Amphiphysin	Stiff-person syndrome, myelopathy and myoclonus, encephalonus	Breast SCLC	95
Ri	Brainstem encephalitis, opsoclonus myoclonus	Breast, SCLC	97
Yo	Cerebellar degeneration	Ovarian, breast	98
Ma2	Limbic encephalitis, brainstem encephalitis	Testicular	96

B, Surface antibodies				

Antibody	Neurological syndromes	CNS pleocytosis (%)	Tumors	Probability of cancer (%)

VGKC	Limbic encephalitis, Morvan's syndrome, Creutzfeld-Jakob disease-like syndrome	41	SCLC, thymoma	31
NMDA	Encephalitis with neuropsychiatric features, catatonia, aphasia, hypoventilation	91	Ovarian, teratoma	9–56
AMPA	Limbic encephalitis, atypical psychosis	90		70
GABA_B_	Limbic encephalitis	80		47
Glycine	Encephalomyelitis, stiff person syndrome	Unknown		3

Adapted from Graus *et al* (2). CNS, central nervous system; SCLC, small cell lung cancer; VGKC, voltage-gated potassium channel; CV2, crossveinless-2; NMDA, N-methyl D-aspartate; AMPA, α-amino-3-hydroxy-5-methyl-4-isoxazolepropionic acid; GABA_B_, γ-aminobutyric acid B.

**Table II.** Analysis of NMDA receptor antibody titers in CSF and serum at admission and during follow-up with respective CSF/serum protein levels and antibody index value.

Patient	Follow-up (months)	Oligoclonal bands	CSF titer	Serum titer	IgG serum (g/l)	IgG CSF (g/l)	CSF titer/protein (mg/l)	Serum titer/protein (g/l)	Antibody index
1	0	Positive	ND	1:1,600	7.67	0.152	NA	80.7	NA
	10		1:1,600	1:51,200	6.11	0.049	32.7	8,379.7	3.9
	11		1:800	1:25,600	2.37	0.014	57.1	10,801.7	5.29
	22		1:400	1:25,600	4.11	0.012	32.3	6,228.7	4.13
	24		1:50	1:1,600	2.83	0.022	2.3	565.4	4.02
2	0	Positive	1:100	1:800	9.68	0.051	2.0	103.3	23.68
	4		1:10	1:400	5.40	0.015	0.7	74.1	9
	7		1:10	1:200	10.10	0.018	0.6	19.8	28.6
3	0	Negative	1:50	Negative	12.10	0.048	1.0	NA	NA
	1		1:10	Negative	8.59	0.023	0.4	NA	NA
4	0	Positive	Negative	1:100	8.86	0.156	NA	11.2	NA
	2		ND	1:800	ND	ND	NA	NA	NA
	7		ND	1:400	5.35	0.041	NA	74.8	NA
5	0	Positive	1:100	1:800	ND	ND	NA	NA	NA
	1		ND	1:800	ND	ND	NA	NA	NA
	2		ND	1:800	ND	ND	NA	NA	NA
6	−21		ND	1:10	ND	ND	NA	NA	NA
	0	Positive	ND	1:100	ND	ND	NA	NA	NA
	6		ND	1:100	ND	ND	NA	NA	NA
	7		ND	1:800	ND	ND	NA	NA	NA
	10		ND	1:50	ND	ND	NA	NA	NA
7	3	Positive	1:200	1:200	12.80	0.097	2.1	15.6	131.55

NMDA, N-methyl D-aspartate; CSF, cerebrospinal fluid; ND, not determined; NA, not available.

**Table III.** Differential diagnoses, common clinical features and useful diagnostic methods to separate these diagnoses from anti-NMDA receptor encephalitis.

Differential diagnosis	Clinical presentation	Diagnostic key
Anti-NMDA encephalitis	Phase 1: Prodromal stage (headache, fever). Phase 2: Behavioral changes, seizures, fluctuating vigilance, hypoventilation. Phase 3: movement abnormalities, vegetative dysfunction	NMDA antibodies in CSF or serum (15% false negative)
Other autoimmune encephalitis	Depending on antibody: Behavioral changes, seizures, amnesia, cerebellar degeneration, Morvan's syndrome, stiff person syndrome	Anti-neuronal antibodies, SCLC, thymoma, breast, ovarian or testicular cancer
Infectious encephalitis (HSV, VZV, syphilis, HIV)	Fever, seizures, focal deficits,	Identification of pathogen (PCR), triphasic waves in CJD
Metabolic encephalopathy	Cognitive dysfunction, reduced vigilance, flapping tremor	Hyperammonemia, uremia, electrolyte imbalance, MRI T2 white matter lesions
Wernicke's encephalopathy	Cognitive dysfunction, reduced vigilance, ocular motor disturbances, ataxia	MRI T2 lesions of the corpora mamillaria, response to thiamin
SREAT (Hashimoto's encephalopathy)	Psychosis, seizures, cognitive dysfunction, reduced vigilance, focal deficits, ataxia	TPO and MAK antibodies, response to corticosteroids
Vasculitis (lupus erythematosus, Sjögren's syndrome)	Fever, myalgia, focal/multifocal deficits,	Ischemic MRI lesions, autoantibodies: dsDNA, SS-A/Ro, SS-A/LA, PR3, MPO
Intoxication	Drug-dependent psychosis, reduced vigilance	Drug screening

NMDA, N-methyl D-aspartate; CSF, cerebrospinal fluid; SCLC, small cell lung cancer; HSV, herpes simplex virus; VZV, Varicella-zoster virus; HIV, human immunodeficiency virus; PCR, polymerase chain reaction; CJD, Creutzfeldt-Jakob disease; MRI, magnetic resonance imaging; SREAT, steroid responsive encephalopathy associated with autoimmune thyroiditis; TPO, thyroid peroxidase; MAK, male germ cell-associated kinase; dsDNA, double-stranded DNA; SS-A, Sjörgen's syndrome-related antigen; PR3, proteinase 3; MPO, myeloperoxidase.
